# Understanding the rapid increase in life expectancy in shanghai, China: a population-based retrospective analysis

**DOI:** 10.1186/s12889-018-5112-7

**Published:** 2018-02-14

**Authors:** Hanyi Chen, Lipeng Hao, Chen Yang, Bei Yan, Qiao Sun, Lianghong Sun, Hua Chen, Yichen Chen

**Affiliations:** Department of Cancer, Injury Prevention and Vital Statistics, Shanghai Pudong New Area Center for Disease Control and Prevention, Fudan University Pudong Institute of Preventive Medicine, Shanghai, 200136 China

**Keywords:** Life expectancy, Joinpoint regression analysis, Cause of death

## Abstract

**Background:**

Life expectancy at birth (LE) is a comprehensive measure that accounts for age-specific death rates in a population. Shanghai has ranked first in LE in China mainland for decades. Understanding the reasons behind its sustained gain in LE provides a good reflection of many other cities in China. The aim of this study is intended to explore temporal trend in age- and cause-specific gains in LE in Shanghai and the probable reasons lay behind.

**Methods:**

Joinpoint regression was applied to evaluate temporal trend in LE and the long time span was then divided accordingly. Contributions to change in LE (1973–2015) were decomposed by age and cause at corresponding periods.

**Results:**

LE in Shanghai could be divided into four phases ie., descent (1973–1976), recovery (1976–1998), rapid rise (1998–2004) and slow rise (2004–2015). The growing LE was mainly attributed to reductions in mortality from the elderly populations and chronic diseases such as cerebrovascular disease, chronic lower respiratory disease, and gastrointestinal cancers (stomach, liver and esophageal cancer).

**Conclusions:**

The four-decade sustained gain in LE in Shanghai is due to the reductions in mortality from the elderly and chronic diseases such as cerebrovascular disease, chronic lower respiratory disease, and gastrointestinal cancers. Further growth momentum still comes from the elderly population.

**Electronic supplementary material:**

The online version of this article (10.1186/s12889-018-5112-7) contains supplementary material, which is available to authorized users.

## Background

Life expectancy at birth (LE) is a comprehensive measure reflecting health conditions of a certain country (or region). LE equals the average years a person is expected to live based on the overall mortality level of a population, hence, is a type of demographic data most commonly used to portray the social and health conditions, as well as well-being of a society. [1, 2].

In October 2016, the Chinese government implemented “healthy China 2030”, a program of action to promote a healthier China in the next 15 years. Program aims include incorporating health into all policies, ensuring individual’s health in an all-round and full cycle, and improving health and health equity. An additional goal is to increase the national LE to 79 by 2030 [3].

China has made extraordinary progress on LE since its founding in 1949. LE has more than doubled, from 35 years before 1949, to 76.3 in 2015 [4]. Increases in LE is often attributed to advancement in public health in China, as well as throughout the world [5–7]. Among all the provinces, Shanghai ranks first in LE [8]. LE in Shanghai is 6.5 years ahead of the national average, reaching to 82.8 in 2015 [9, 10].

Shanghai has taken lead in LE for 12 years nationwide [11]. Temporal trend of LE of the forerunner may help find both common and special reasons behind a rapid increase and long-term-leading position. Furthermore, in view of the shift from infectious diseases to non-communicable causes occurring nationwide, understanding LE in Shanghai also provides a good reflection of the future in many other cities in China, which makes sense to achieve the goal of “healthy China 2030” [12, 13].

Since comprehensive Mortality Registration System in Shanghai dates back to 1973 [14], our study is intended (1) to investigate change in LE in Shanghai between 1973 and 2015; (2) to explore age-, and cause- specific difference in LE.

## Methods

### Data sources

Although the 42-year information on detailed causes of death for Shanghai as a whole was unavailable, LE and individual mortality data were collected from Pudong New Area instead. Pudong New Area accounts for one-fifth of the total population in Shanghai, consisting of both urbanized and rural places [15]. Pudong has been oriented as an economic and technological development zone since early 1990s. Hence, it is the microcosm of China’s reformation and a good representative of Shanghai.

The Mortality Registration System of Pudong New Area, covers medical institutions of all levels and data is checked against local population registry on a monthly basis. Periodic evaluations, data cleaning and compilation have been done at both the county and provincial levels according to standard guidelines [16]. The infant death reporting system has been established in local hospitals since 1974 [14]. The funeral and cremation system has been implemented throughout the city since 1980s [14]. All these measures ensure the completeness of the registration system to the maximum extent.

### Coding and conversion

As the data covered a long time span, the 42-year data was divided into three parts according to coding methods. Data for 1992–2001 and 2002–2015 were coded based on the International Classification of Diseases, 9th and 10th Revision (ICD-9 and ICD-10), respectively. Historical records before 1992 had been in paper form and were digitized and recoded based on ICD-10. The recoded historical data was checked against historical annual reports by cause and age. In case of a difference in rules for selecting underlying cause of death (UCOD), UCOD was modified according to ICD-10 to avoid inconsistency in classification. All the data was then analyzed according to the Chinese Classification of Diseases (CCD), due to previous use in Shanghai’s annual report and assisted to bridge ICD-9 and ICD-10.

All causes of death were coded by rigorously trained clinicians, and each record was further verified by local Center for Disease Control and Prevention (CDC). Review of medical records, reports from family members, or police records were carried out if there were any discrepancies. Ill-defined data was greatly reduced through above- mentioned methods.

### Analyses

Joinpoint regressions have been widely conducted to explore potential changes in trends [17, 18]. The basic idea is to model the time series using a few continuous linear segments [19]. The method follows the principle of minimization of the weighted sum of squared errors and the choice of the number of joinpoints is on the basis of permutation tests [20]. Compared to other regression methods used to investigate trends to find the best-fit line through years of data, the joinpoint analysis test whether a multi-segmented line is a significantly better fit than a straight or less-segmented line [21, 22]. Meanwhile, joinpoint analysis provides a much clearer picture of what is happening during a distinct period than a single summary trend statistic [23].

Therefore, the study introduced joinpoint regression to scientifically evaluate a temporal trend in LE during the 42 year period. The method identifies whether there are statistically significant differences in the trend of LE and quantifies the speed with which the increase or decrease occurs. The year was assigned as an independent variable while the annual LE is a dependent variable. The maximum number of joinpoints was set at 5 and Heteroscedastic Errors Option was set at constant variance. The Grid Search method was selected and Monte Carlo simulation was performed with the number of permutations set to 4499 to determine the optimal number of change-points in segmented line regression.

LE was estimated on the basis of annual abridged life tables, which were constructed with reference to a standard table [14]. The age intervals of the life table were age 0–1, 1–4, 5–9, and in subsequent five-year age groups up to age 85 (80 for historical data) with an open end. Details of the life table and relative equations could be referred [24, 25]. Contributions to LE were computed and expressed in years gained or lost at corresponding periods using the decomposition method [26]. The method enables the separation of differences in LE into factors related to age and cause of death [27]. Hence, helping to demonstrate the significance of a particular cause of death on the change in years of life lost. Reductions in mortality from specific age groups or causes would make positive contributions to the change in LE, and vice versa.

### Data quality and sensitivity analysis

Given the potential variation of data quality, the quality of data was evaluated across different years by a recommended method [28]. The method defined and classified garbage codes into different types to help assess data quality and fully understand the problem. Based on the method, the number and type of garbage codes were analyzed and the comparability of data throughout different years was enhanced. Cause-deletion method was performed as a sensitivity analysis to determine how the results were influenced by the amount of change attributed to other causes of death [29].

Statistical analysis was performed with Stata/SE 11.0 (College Station, TX). Joinpoint regression analyses were carried out using Joinpoint Regression Program, Version 4.0.4 (US National Cancer Institute, MD).

## Results

### Trend of life expectancy

**Fig. 1:**
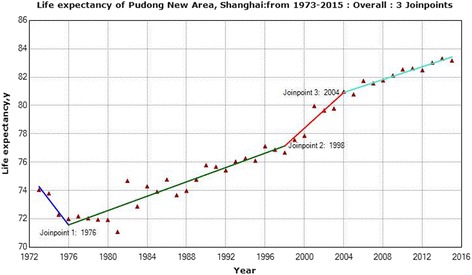
Trend in life expectancy of Shanghai Pudong, from 1973 to 2015. Life expectancy varied within the range between 72 and 83. Three joinpoints were found ie., year 1976, 1998 and 2004

Between 1973 and 2015, LE increased from 74.1 to 83.2 and this trend could be divided into four phases. The descent (1973–1976) saw LE dropped dramatically from 74.1 to 72.0 (slope is − 0.9 with 95% CI (− 1.7, − 0.1)). LE stepped into a recovery phase (1976–1998), increasing steadily from 72.0 to 76.7 (slope is 0.3 with 95% CI (0.2, 0.3)). A sharp increase was observed during 1998–2004 (slope is 0.6 with 95% CI (0.3, 1.0)). After then, trend in LE turned into a slower increase and reached to 83.2 in 2015 (slope is 0.2 with 95% CI (0.1, 0.3)) (Table 1 and Fig. 1).

**Table 1:** Joinpoint regression analysis of life expectancy in Shanghai Pudong New Area, 1973–2015

Phase	Trend period	Difference	Slope (95%CI)	*Z*
Descent	1973–1976	−2.05	−0.903* (−1.727 – − 0.078)	−2.146
Recovery	1976–1998	4.68	0.253*** (0.211–0.295)	11.816
Rapid rise	1998–2004	4.31	0.630** (0.261–0.998)	3.346
Slow rise	2004–2015	2.19	0.228*** (0.117–0.339)	4.016

*P-value< 0.05; **P -value< 0.01; ***P -value< 0.001

### Age-specific contributions to life expectancy at different phases

**Fig. 2:**
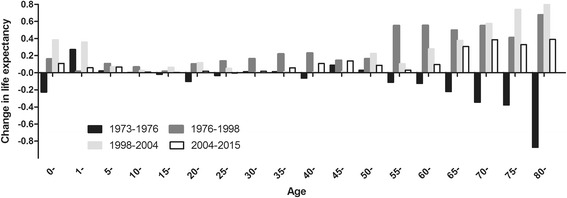
Age-specific contributions to life expectancy in different phases. Infant group was separately listed. Each bar indicated net contribution of mortality changes to the total life expectancy in different phases

At descent phase, negative contributions to LE were observed at the age < 1, 15–29, 40–44, and aged 55 and older. LE started to increase in 1976 and all age groups contributed positively. Figure 2 showed that residents aged 55 and older contributed most to the total longevity increase during 1976–2015, especially for age group of 80 years and older.

Proportions of contribution to LE varied in age groups over time. At recovery phase, aged 0–14, 15–54 and 55 and older accounted for 7.35%, 24.64% and 68.01% of gains in LE, respectively. The above-mentioned age groups accounted for 17.85%, 10.69%, 71.47%, and 10.93%, 19.31%, 69.76% of gains in LE at the subsequent rise phases, respectively.

### Disease group-specific contributions to life expectancy at different phases

Gains and loss of LE attributed to different disease groups were presented. At descent phase, decrease in LE mainly resulted from respiratory system diseases (− 0.8 years; 38.86%), cardiovascular diseases (− 0.8 years; 37.09%), cancer (− 0.4 years; 20.81%), perinatal deaths (− 0.4 years; 17.57%), external causes (− 0.2 years; 8.11%) and digestive system diseases (− 0.1 years; 5.53%).

At the recovery phase, increases in LE mainly resulted from respiratory system diseases (1.0 years; 21.33%), external causes (0.6 years; 13.61%), digestive system diseases (0.6 years; 13.51%), cardiovascular diseases (0.6 years; 11.63%), infectious diseases (0.4 years; 8.81%), and cancer (0.4 years; 7.63%). The six disease groups accounted for over 76% of the total increase in LE.

For the rapid rise, cardiovascular diseases outreached respiratory system diseases and became the leading contribution to the increase in LE (1.7 years, 40.65% versus 1.1 years, 27.01%). Followed by cancer (0.3 years; 7.92%), congenital malformations (0.3 years; 6.78%), perinatal deaths (0.3 years; 6.03%) and external causes (0.2 years; 4.95%). The six disease groups accounted for over 93% of the total increase in LE.

**Fig. 3:**
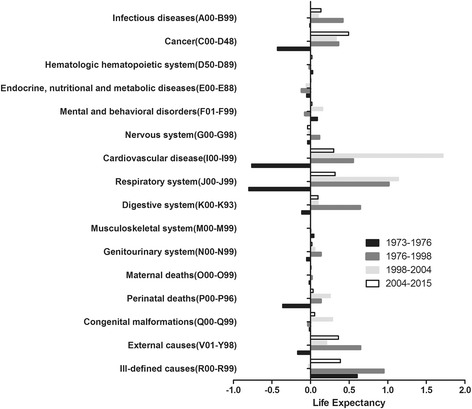
Disease group-specific contributions to life expectancy. All disease group codes were from ICD-10. Each bar indicated net contribution of mortality changes to the total life expectancy in different phases

For the slow rise, increases in LE mainly resulted from cancer (0.5 years; 22.42%), external causes (0.4 years; 16.41%), respiratory system diseases (0.3 years; 14.49%), cardiovascular diseases (0.3 years; 13.70%), infectious diseases (0.1 years; 6.10%) and digestive system diseases (0.1 years; 4.42%). The six disease groups accounted for over 77% of the total increase in LE (Fig. 3).

### Cause-specific contributions to life expectancy at different time

The leading ten causes of death fall into the above-mentioned main disease groups at corresponding phases. At descent phase, the leading ten causes which contributed negatively to LE were chronic lower respiratory disease, cerebrovascular disease, external causes (drowning and self-harm), cancer (stomach cancer, lung cancer, colon and rectum cancer, liver cancer), chronic rheumatic heart disease, and neonatal asphyxia and trauma.

Chronic lower respiratory disease took the lead in contribution to increase in LE at recovery phase, followed by respiratory tuberculosis, cirrhosis, self-harm, pneumonia, cancer (stomach cancer, liver cancer and esophagus cancer), and cardiovascular diseases (pulmonary heart disease and chronic rheumatic heart disease). These ten causes accounted for 63.6% of the total increase at that time.

For the rapid rise, cerebrovascular disease overtook chronic lower respiratory disease and became the leading cause of contribution. Neonatal asphyxia and trauma, ischemic heart disease, congenital heart anomalies, cancer (liver cancer, stomach cancer and esophagus cancer), poisonings, and hepatitis also made great contributions. As a result, up to 82.0% of the total increase was attributed to these diseases.

**Fig. 4:**
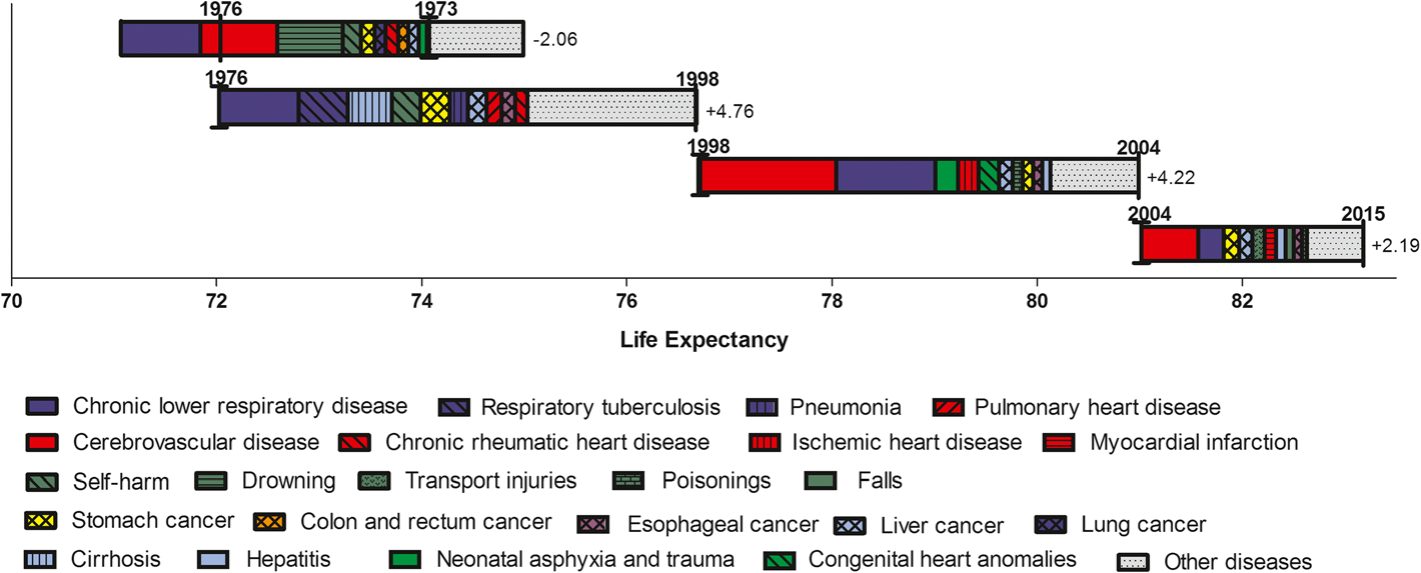
Cause-specific contributions to life expectancy in different phases. Specific causes were classified according to CCD

For the slow rise, the two leading causes remained same, ie., cerebrovascular disease and chronic lower respiratory disease. In addition, contributions of LE from the three leading cancer (stomach cancer, liver cancer, and esophagus cancer) improved. External causes (transport accidents, falls, and poisoning) also made contributions, accounting for over 11% of increase in LE. All the ten causes contributed to 74.7% of the total increase in LE (Fig. 4).

## Discussion

A major focus of the research is to investigate age- and cause- specific change in LE in Shanghai between 1973 and 2015. We quantified age- and cause- specific contributions to LE using Arriaga’s decomposition method in this study [26]. Our findings demonstrate that the growing LE in Shanghai was mainly attributed to the reduction of mortality among the elderly, rather than infants. Important contributions to the gain were made by constant reductions in mortality from cerebrovascular disease, chronic lower respiratory disease, and gastrointestinal cancers (stomach, liver and esophageal cancer).

Our finding in that the reduction in infant mortality neither played the most important role, nor could be neglected was different from previous findings in some Asian and western countries [6]. Reductions in infant mortality represented the largest age-group contribution to increase in LE in other Asian countries, but this was different in Shanghai. The major driving force of continuous gains in LE came from the elderly. Shanghai entered the ageing society in early 1980s. There appeared to be a trend that the contribution to LE, centralized more from advanced age (65 years and older), which was consistent in other high-income countries [30]. Epidemiological transition from high mortality to low mortality dominated by non-communicable diseases, may explain the shift of contribution from median age to advanced age.

Increases in LE rarely benefited from a reduction in infant mortality since 1990s in Western countries. However, contribution to LE from infant mortality was positive since the recovery phase in Shanghai. Significant contribution appeared in the rapid rise phase, which might be attributed to effective promotion in pediatric healthcare, including implementations of family planning, immunization, and nutritional support [31]. It is worth noting that contribution to LE from infant mortality dropped sharply in the slow rise phase, which was somehow in line with findings in Western countries, but in a later period. Given the relatively low infant mortality, further improvements in infant mortality will be difficult to achieve [32].

The worldwide improvements in medicine and public health may explain why major contributions to the increase in LE were from non-communicable diseases (cerebrovascular disease, chronic lower respiratory disease and cancers). Cardiovascular diseases, represented by cerebrovascular disease, led the contribution to LE in Shanghai recent years [33]. Since prevalence of risk factors such as obesity and hypertension increased, declined mortality was more likely to be attributed to improvements in access to and quality of medical care and treatment of cerebrovascular diseases [34]. However, improved survival of patients with coronary heart diseases were less prominent, compared with those of cerebrovascular disease. Given that approximately 40% of deaths came from cardiovascular diseases in China each year, and the dramatic decline of cardiovascular diseases brought Japan the longest LE in the world [35], there is still a large potential for further improvements in primary and secondary prevention of cardiovascular disease in Shanghai [32, 35, 36].

Consistent with findings in developed countries, our data showed a strong and continuous contribution from declining mortality of stomach, esophageal, and liver cancer [37]. Improvements in public sanitation (centralized drinking water supply system), life style (less cured food and more fresh vegetables) and the control of *Helicobacter pylori* and other parasites (schistosome) infections were potential causes for the reduction of the incidence [38–42].

Lung cancer contributed little, given the fact that it ranked first in mortality in cancer. Moreover, colorectal cancer and pancreatic cancer showed increase tendencies of mortality [43, 44]. Cancers are caused by a complex relationship of lifestyle, socioeconomic, and genetic factors. Further decrease in malignant neoplasms would be possible both by improving primary and secondary preventions. Efforts on behaviors modification such as dietary habits, tobacco use, physical activity, and social support should always be unremitting [45]. Screening programs were also expected to achieve a further reduction in mortality from malignancies.

As a microcosm of China, Shanghai also witnessed the huge impact of political and social factors on LE. LE set back by over 2 years during 1973 to 1976. A majority of disease groups made negative contribution to LE. Among them, external causes represented by self-harm is particularly prominent. Drowning and self-harm were the top five negative contributors to LE during the descent phase, but self-harm turned to be the fourth positive contributors to LE at the recovery phase. The Cultural Revolution in China (1966–1976) left a scar in Chinese history. Social unrest, distorted moral values, and repressed emotion led to extremes. As a result, the contribution of self-harm on LE differed greatly during the descent and recovery phase. Changes of political and social environments strongly influence LE, as proven in other countries [32, 46]. A peaceful and stable social and political environment of China was the foundation of continuous increase in LE and also the underlying reason for the outstanding LE of Shanghai.

The top 10 disease groups of death for China and Shanghai were almost same in rank and type (Additional file 1: Appendix Table A2). Shanghai shares the same experience with the shift from infectious diseases to non-communicable causes as is throughout China; however, Shanghai also has its specialty. Shanghai was divided into the first group by Zhou, with low mortality even by the standards of high-income countries.^8^ However, Shanghai did not rank first in GDP per capita among the provinces in the first group in China. Reasons for its high LE is likely due to its long-term emphasis on public health. Shanghai is one of the earliest and best provinces to implement the strategy of modern TB (tuberculosis) control in China [47]. Its average positive rate of schistosomiasis was 20.5% by stool examination and positive rate of hepatitis B surface antigen (HBsAg) in newborns 43.6% [47–49]. With the implementation of a wide range of prevention and control measures, such as schistosomiasis control programs, hepatitis B vaccinations, and infectious disease surveillance, diseases had been eliminated or greatly reduced in the mid-1980s [48, 50, 51]. All the above-mentioned facts corroborate Shanghai’s tremendous improvements in infectious and digestive diseases, especially at the recovery phase (1976–1998) and for cirrhosis and hepatitis.

Our study had several limitations. First, the quality of data varied in different periods, which might affect the accuracy of the estimated trends in causes of death. Since the full coverage of the Mortality Registration System began in early 1973 and historical data was recoded based on the ICD-10 to avoid classification inconsistencies, we believe the 42-year span of data was comparable in general.

Second, the characteristics of the population at different times should be examined fully to exclude potential influence on LE from population churn. Given that the population we studied was registered Shanghai residents, potential influence of migration was little as shown in the supplementary materials.

Third, CCD was not precise compared with causes of death based on the Global Burden of Disease (GBD) study [8]. CCD is widely accepted in China over the decades and helped to keep comparability between data, however, CCD has its limitations, for example, ill-defined causes in our study were basically centralized in R code. The impact of ill-defined causes to our results concentrated in the aged group, and the nearer the year, the less impact on the results. We will attempt to classify causes of death on the basis of GBD rules in future.

In addition, the population was analyzed as a whole in this study without distinction between male and female. In our view, the overall impact on human from achievements in medicine and public health, plus the political and social factors were approximately same across both sexes. However, we admitted that there were gender differences, especially in specific diseases involved. We will explore this issue in greater detail in a future article (Additional file 2).

## Conclusion

LE in China increases rapidly, and the shift from infectious diseases to non-communicable causes occurs nationwide. Among its entire provinces, Shanghai ranks first and continues to be in the lead. From the 42-year retrospective study, LE in Shanghai could be divided into four phases. The growing LE was mainly attributed to reductions in mortality from the elderly and chronic diseases such as cerebrovascular disease, chronic lower respiratory disease and gastrointestinal cancers (stomach, liver and esophageal cancer). Reduction in infant mortality also plays an important role, especially in the recovery and rapid rise phase; however, its impact dropped sharply in the slow rise phase. Coronary heart diseases and lung cancer showed less contribution to LE, compared with cerebrovascular disease and gastrointestinal cancers. Political and social factors are critical for analysis as well. LE in Shanghai set back by over 2 years during 1973 to 1976, particularly due to external causes represented by self-harm. Understanding the LE of the forerunner will have reference value for many other cities in China. Age- and cause- specific decomposition of LE assist in determining key population and diseases, set specific goal for further achievement in LE, and ensure realization of “Healthy China 2030”.

In conclusion, the four-decade sustained gain in LE in Shanghai is attributed to the reductions in mortality from the elderly population and chronic diseases, such as cerebrovascular disease, chronic lower respiratory disease, and gastrointestinal cancers. Further growth momentum in LE continues to stem from the elderly population.

## Additional files

Additional file 1: Table A1. List of causes of death based on CCD (Chinese Classification of Diseases) and the corresponding ICD-10 codes. **Table A2.** Top 10 Causes of death of the nation, province and study area in 2015. **Table A3.** The sensitivity analysis of Arriaga’s decomposition method. **Table A4** The characteristics of the population at different time. (DOCX 29 kb)

Additional file 2: Figure S1 A1. Percentage of all deaths coded to garbage codes by age in different years. (TIFF 1817 kb)
