# Supplementary material for: Understanding the rapid increase in life expectancy in shanghai, China: a population-based retrospective analysis
Source: BMC Public Health. 2018 Feb 14;18:256. doi: 10.1186/s12889-018-5112-7 (PMC5813363; doi:10.1186/s12889-018-5112-7)
Supplement: Supplementary file 1 — List of causes of death based on CCD (Chinese Classification of Diseases) and the corresponding ICD-10 codes. Table A2. Top 10 Causes of death of the nation, province and study area in 2015. Table A3. The sensitivity analysis of Arriaga’s decomposition method. Table A4 The characteristics of the population at different time. (DOCX 29 kb) [file 12889_2018_5112_MOESM1_ESM.docx]

**Additional file 1**

**Appendix Table 1** List of causes of death based on CCD (Chinese Classification of Diseases) and the corresponding ICD-10 codes

| CCD Cause | | | ICD-10 Code |
| --- | --- | --- | --- |
| Infectious and parasitic diseases | **A00-B99** | | |
| Typhoid and paratyphoid fevers | | A01.0-A01.4 | |
| Shigellosis | | A03.0-A03.9 | |
| Other bacterial intestinal infections and salmonellla infections | | A02.0-A02.9,A04-A09 | |
| Respiratory tuberculosis | | A15-A16 | |
| Other tuberculosis | | A17-A19 | |
| Leptospirosis | | A27 | |
| Tetanus neonatorum and obstetrical tetanus | | A33-A35 | |
| Whooping cough | | A37 | |
| Meningococcal infection | | A39 | |
| Mosquito-borne viral encephalitis | | A83 | |
| Other viral haemorrhagic fevers | | A98.5 | |
| Measles | | B05 | |
| Viral hepatitis | | B15-B19 | |
| HIV | | B20-B24 | |
| Malaria | | B50-B54 | |
| Schistosomiasis | | B65,B94 | |
| Neoplasms | | **C00-D48** | |
| Nasopharynx cancer | | C11 | |
| Esophageal cancer | | C15 | |
| Stomach cancer | | C16 | |
| Colon and rectum cancer | | C18-C21 | |
| Liver cancer | | C22 | |
| Tracheal, bronchus and lung cancers | | C33-C34 | |
| Breast cancer | | C50 | |
| Cervical cancer | | C53 | |
| Bladder cancer | | C67 | |
| Leukemia | | C91-C95 | |
| Diseases of the blood/blood-forming organs and immune system | | **D50-D89** | |
| Anemia | | D50-D53,D55-D64 | |
| Endocrine, nutritional and metabolic diseases | | **E00-E88** | |
| Diabetes mellitus | | E10-E14 | |
| Nervous system | | **G00-G98** | |
| Meningitis | | G00-G03 | |
| Cardiovascular diseases | | **I00-I99** | |
| Acute rheumatic fever | | I00-I02 | |
| Rheumatic heart diseases | | I05-I09 | |
| Hypertensive heart disease | | I11 | |
| Acute myocardial infarction | | I21 | |
| Ischaemic heart diseases | | I20-I25（except for I21) | |
| Pulmonary heart diseases | | I26-I27 | |
| Cerebrovascular disease | | I60-I69 | |
| Respiratory system | | **J00-J99** | |
| Pneumonia | | J12-J18.9 | |
| Chronic lower respiratory diseases | | J40-J47 | |
| Pneumoconiosis | | J60-J65 | |
| Digestive system | | **K00-K93** | |
| Gastric ulcer | | K25-K27 | |
| Appendix | | K35-K37 | |
| Intestinal obstruction | | K56 | |
| Cirrhosis | | K70-K76 | |
| Genitourinary system | | **N00-N99** | |
| Glomerular diseases and renal tubulo-interstitial | | N00-N15 | |
| Maternal deaths | | **O00-O99** | |
| Abortive outcome | | O00-O07 | |
| Oedema hypertensive disorders | | O10-O16 | |
| Obstructed labor | | O64-O66 | |
| Postpartum haemorrhage | | O72 | |
| Perineal laceration during delivery and other obstetric trauma | | O70-O71 | |
| Complications predominantly related to the puerperium | | O85-O92 | |
| Perinatal deaths | | **P00-P96** | |
| Disorders related to length of gestation and fetal growth | | P05-P07 | |
| Birth trauma and asphyxia | | P10-P15,P21 | |
| Haemolytic disease of fetus and newborn | | P55-P57 | |
| Other conditions of integument specific to fetus and newborn | | P83 | |
| Congenital malformations | | **Q00-Q99** | |
| Congenital heart diseases | | Q20-Q24 | |
| External causes | | **V01-Y98** | |
| Transport injuries | | V00-V99 | |
| Accidental poisoning by and exposure to noxious substances | | X40-X49 | |
| Falls | | W00-W19 | |
| Exposure to smoke, fire and flames | | X00-X06.9,X08-X09 | |
| Exposure to forces of nature | | X30-X39.9 | |
| Accidental drowning and submersion | | W65-W70.9,W73-W74.9 | |
| Accidental threats to breathing except for inhalation of gastric or ingestion food | | W75-W77,W81-W84 | |
| Struck by thrown, projected or falling object | | W20 | |
| Contact with sharp things or machinery | | W25-W31 | |
| Exposure to electric transmission | | W85-W87 | |
| Self-harm | | X60-X84 | |
| Assault | | X85-Y09 | |

**Appendix Table 2** Top 10 Causes of death of the nation, province and study area in 2015

| Rank | China^a^ | |  | Shanghai^b^ | |  | Pudong | |
| --- | --- | --- | --- | --- | --- | --- | --- | --- |
|  | Cause | % |  | Cause | % |  | Cause | % |
| 1 | Cardiovascular | 42.6 |  | Cardiovascular | 39.3 |  | Cardiovascular | 38.0 |
| 2 | Cancer | 26.4 |  | Cancer | 30.7 |  | Cancer | 31.2 |
| 3 | Respiratory | 11.8 |  | Respiratory | 9.5 |  | Respiratory | 9.6 |
| 4 | External | 6.1 |  | Endocrine | 5.0 |  | Endocrine | 5.0 |
| 5 | Endocrine | 3.1 |  | External | 4.7 |  | External | 4.9 |
| 6 | Digestive | 2.3 |  | Digestive | 2.3 |  | Digestive | 2.2 |
| 7 | Nervous | 1.1 |  | Nervous | 1.4 |  | Nervous | 1.3 |
| 8 | Infectious | 1.1 |  | Infectious | 1.1 |  | Infectious | 1.1 |
| 9 | Genitourinary | 1.1 |  | Mental | 1.1 |  | Mental | 0.9 |
| 10 | Mental | 0.5 |  | Genitourinary | 0.8 |  | Genitourinary | 0.6 |

1. China Statistical Yearbook
2. Shanghai Statistical Yearbook

**Appendix Table 3** The sensitivity analysis of Arriaga’s decomposition method

| **Cause of death** | **Change of the gain from cause-deletion method** | **Gain in e0 from Arriaga’s method** | **Difference**^a^ |
| --- | --- | --- | --- |
|  | **1973-1976** | | |
| **Cardiovascular disease** | -0.07 | -0.76 | 0.69 |
| **Cancer** | -0.03 | -0.43 | 0.40 |
| **Respiratory system** | 0.29 | -0.80 | 1.09 |
| **External causes** | 0.03 | -0.17 | 0.20 |
| **Endocrine diseases** | 0.04 | -0.05 | 0.09 |
| **Digestive system** | -0.10 | -0.11 | 0.01 |
| **Infectious diseases** | -0.10 | -0.01 | -0.09 |
| **Perinatal deaths** | 0.35 | -0.36 | 0.71 |
|  | **1976-1998** | | |
| **Cardiovascular disease** | 2.22 | 0.55 | 1.67 |
| **Cancer** | 0.66 | 0.36 | 0.30 |
| **Respiratory system** | 0.12 | 1.01 | -0.90 |
| **External causes** | -0.45 | 0.65 | -1.10 |
| **Endocrine diseases** | 0.30 | -0.12 | 0.42 |
| **Digestive system** | -0.35 | 0.64 | -0.99 |
| **Infectious diseases** | -0.20 | 0.42 | -0.62 |
| **Perinatal deaths** | 0.01 | 0.12 | -0.13 |
|  | **1998-2004** | | |
| **Cardiovascular disease** | 2.00 | 1.71 | 0.29 |
| **Cancer** | 2.16 | 0.33 | 1.83 |
| **Respiratory system** | 0.16 | 1.14 | -0.98 |
| **External causes** | 0.71 | 0.21 | 0.50 |
| **Endocrine diseases** | 0.93 | -0.06 | 0.99 |
| **Digestive system** | 0.69 | 0.10 | 0.59 |
| **Infectious diseases** | 0.61 | 0.10 | 0.51 |
| **Perinatal deaths** | 0.38 | 0.25 | -0.13 |
|  | **2004-2015** | | |
| **Cardiovascular disease** | 2.32 | 0.30 | 2.02 |
| **Cancer** | 0.10 | 0.49 | -0.39 |
| **Respiratory system** | -0.15 | 0.32 | -0.47 |
| **External causes** | -0.26 | 0.36 | -0.62 |
| **Endocrine diseases** | 0.14 | 0.00 | 0.14 |
| **Digestive system** | -0.03 | 0.10 | -0.13 |
| **Infectious diseases** | -0.11 | 0.13 | -0.24 |
| **Perinatal deaths** | -0.02 | 0.03 | -0.05 |

a. difference between cause-deletion and the decomposition method were the amount of change attributable to other causes of death

**Appendix Table 4** The characteristics of the population at different time

| Age group | Percentage | | | |
| --- | --- | --- | --- | --- |
|  | 1976 | 1998 | 2004 | 2015 |
| 0- | 1.14 | 0.36 | 0.60 | 0.76 |
| 1- | 6.86 | 2.34 | 1.76 | 3.45 |
| 5- | 9.94 | 5.59 | 2.76 | 3.58 |
| 10- | 12.60 | 7.03 | 3.72 | 2.78 |
| 15- | 14.47 | 6.51 | 6.46 | 3.04 |
| 20- | 10.39 | 5.04 | 7.82 | 4.15 |
| 25- | 7.93 | 5.91 | 7.28 | 6.85 |
| 30- | 4.80 | 8.31 | 6.20 | 8.49 |
| 35- | 5.10 | 11.50 | 6.22 | 7.87 |
| 40- | 4.95 | 12.25 | 9.31 | 6.48 |
| 45- | 4.59 | 8.83 | 11.89 | 6.61 |
| 50- | 4.06 | 5.17 | 10.31 | 8.32 |
| 55- | 3.23 | 4.22 | 7.06 | 9.94 |
| 60- | 3.10 | 5.30 | 4.18 | 9.65 |
| 65- | 2.91 | 4.37 | 4.06 | 6.61 |
| 70- | 1.98 | 3.35 | 4.35 | 3.72 |
| 75- | 1.22 | 2.15 | 3.11 | 2.94 |
| 80- | 0.74 | 1.78 | 2.94 | 4.76 |
| Total | 100.00 | 100.00 | 100.00 | 100.00 |
