# Supplementary figures and images for: Understanding the rapid increase in life expectancy in shanghai, China: a population-based retrospective analysis
Source: BMC Public Health. 2018 Feb 14;18:256. doi: 10.1186/s12889-018-5112-7 (PMC5813363; doi:10.1186/s12889-018-5112-7)

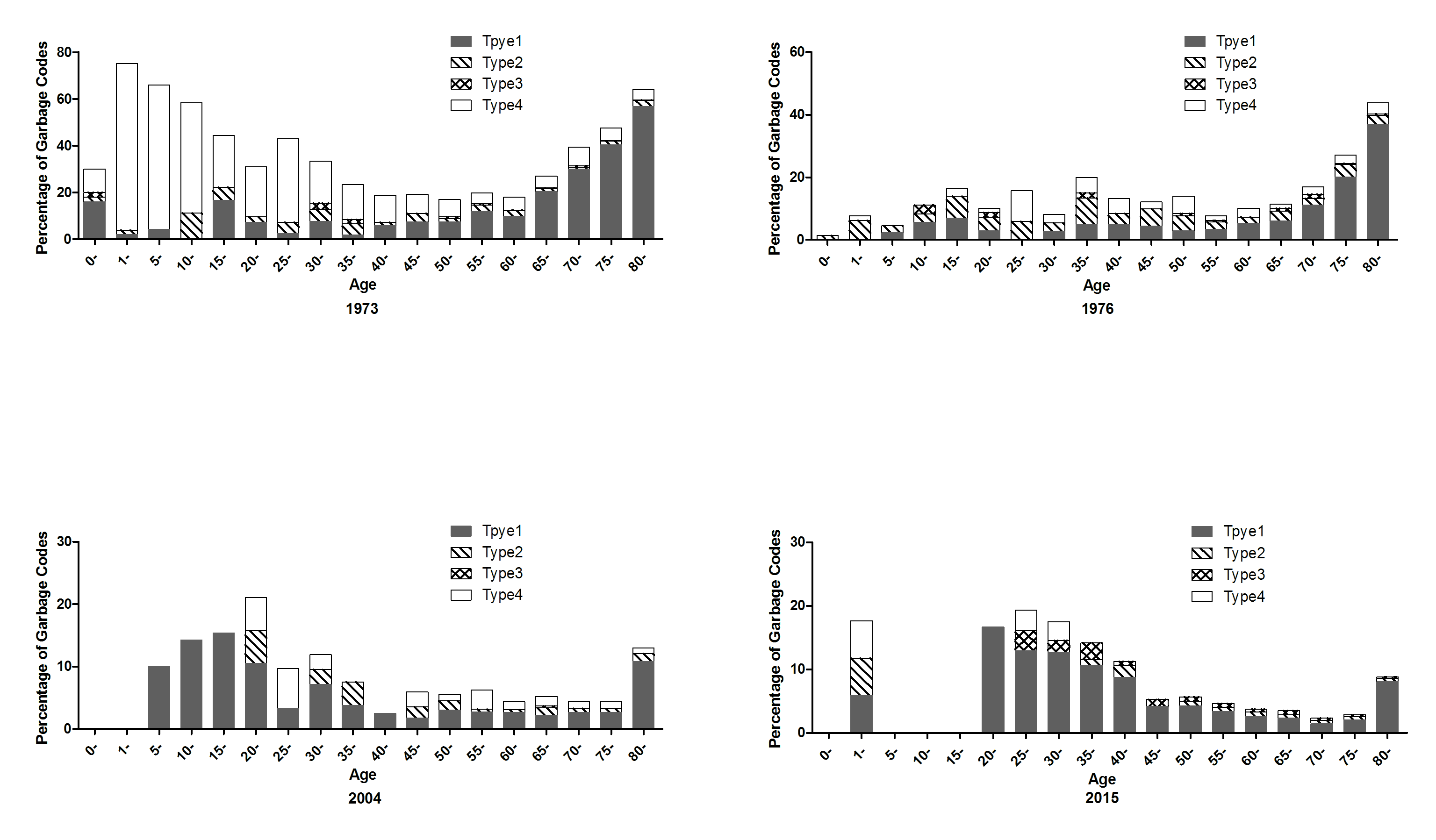

Supplement: Supplementary file 2 — Percentage of all deaths coded to garbage codes by age in different years. (TIFF 1817 kb) [file 12889_2018_5112_MOESM2_ESM.tif]
